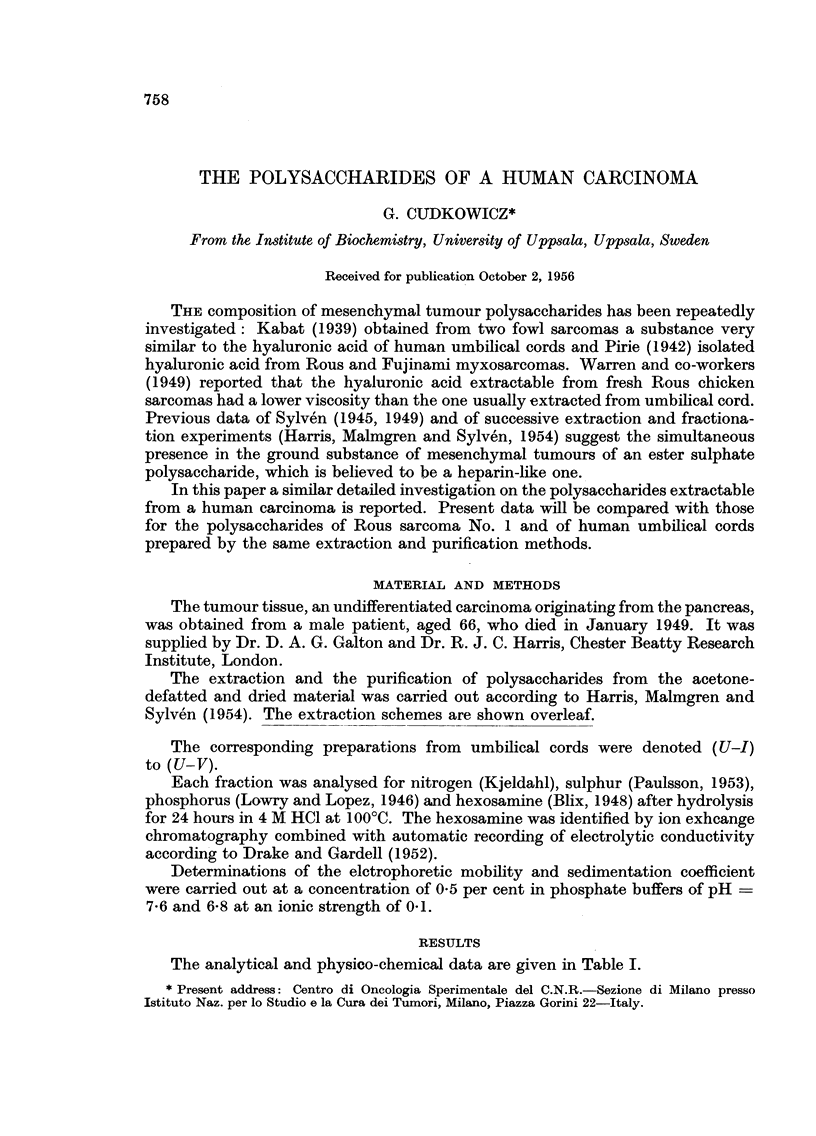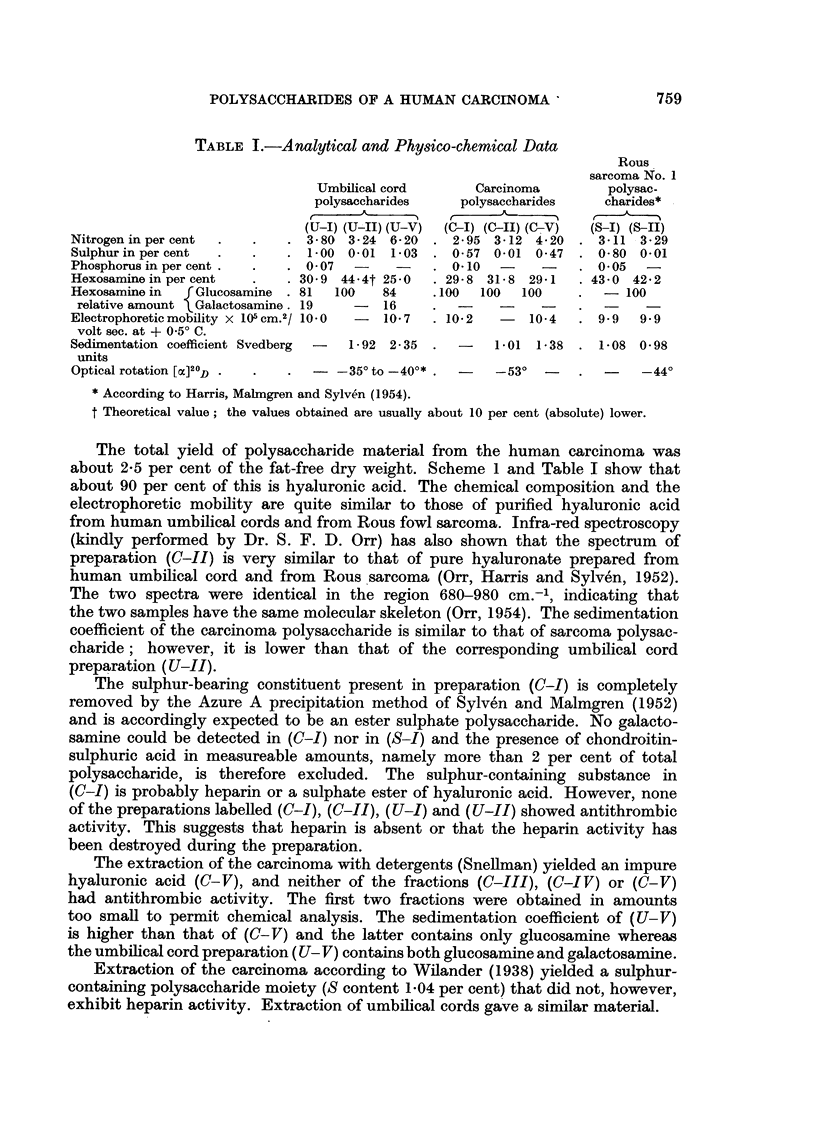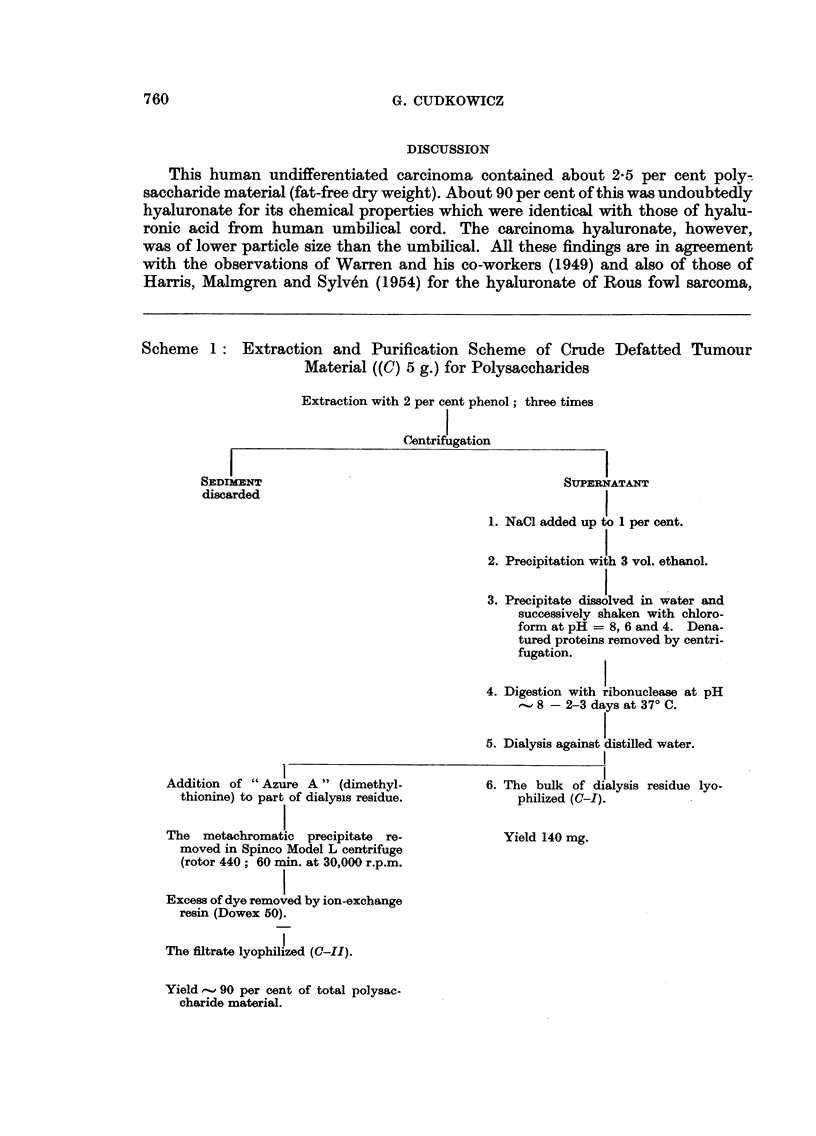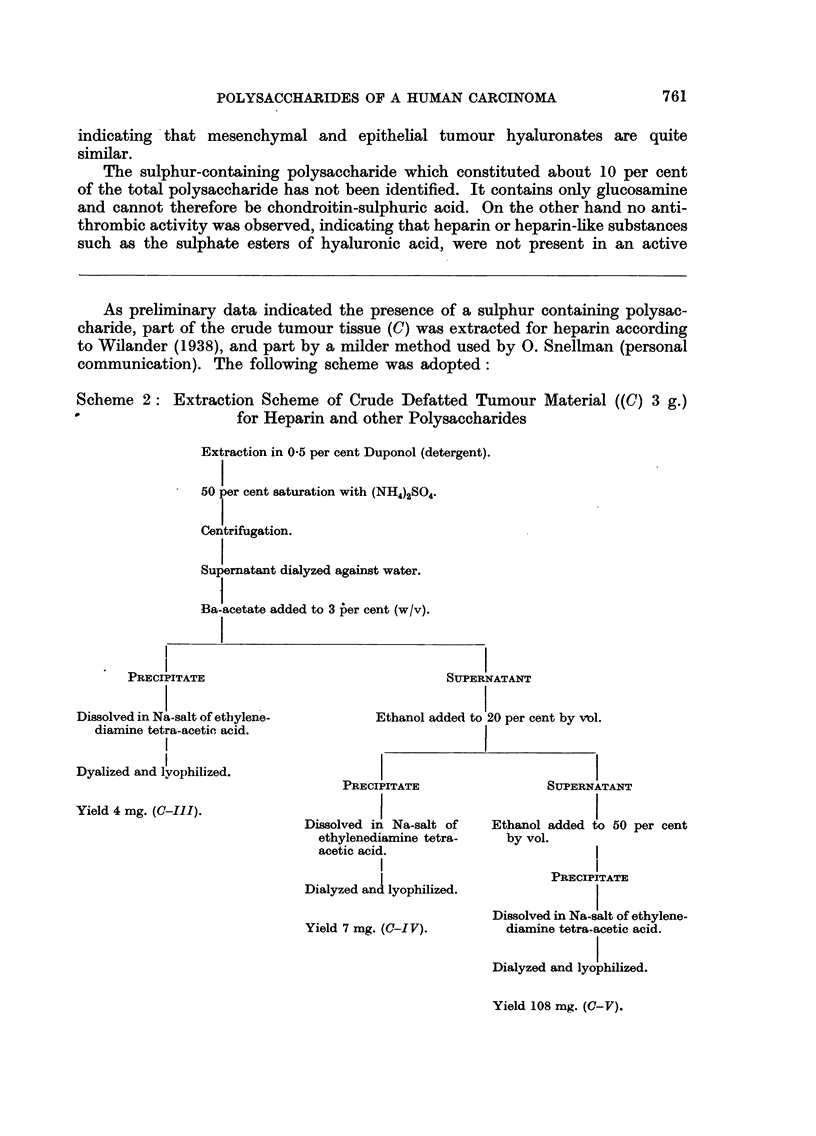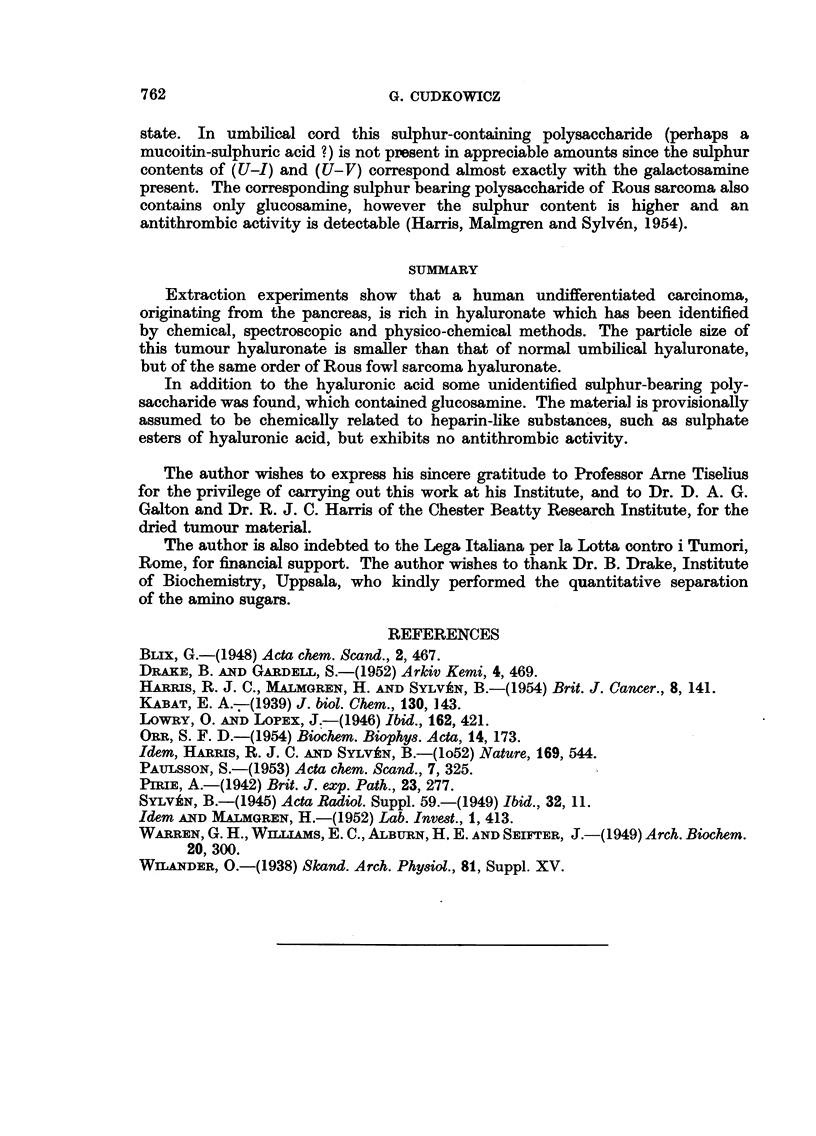# The Polysaccharides of a Human Carcinoma

**DOI:** 10.1038/bjc.1956.92

**Published:** 1956-12

**Authors:** G. Cudkowicz


### THE POLYSACCHARIDES OF A HUMAN CARCINOMA

G. CUDKOWICZ*

*From the Institute of Biochemistry, University of Uppsala, Uppsala, Sweden*

Received for publication October 2, 1956

THE composition of mesenchymal tumour polysaccharides has been repeatedly investigated : Kabat (1939) obtained from two fowl sarcomas a substance very similar to the hyaluronic acid of human umbilical cords and Pirie (1942) isolated hyaluronic acid from Rous and Fujinami myxosarcomas. Warren and co-workers (1949) reported that the hyaluronic acid extractable from fresh Rous chicken sarcomas had a lower viscosity than the one usually extracted from umbilical cord. Previous data of Sylvén (1945, 1949) and of successive extraction and fractionation experiments (Harris, Malmgren and Sylvén, 1954) suggest the simultaneous presence in the ground substance of mesenchymal tumours of an ester sulphate polysaccharide, which is believed to be a heparin-like one.

In this paper a similar detailed investigation on the polysaccharides extractable from a human carcinoma is reported. Present data will be compared with those for the polysaccharides of Rous sarcoma No. 1 and of human umbilical cords prepared by the same extraction and purification methods.

### MATERIAL AND METHODS

The tumour tissue, an undifferentiated carcinoma originating from the pancreas, was obtained from a male patient, aged 66, who died in January 1949. It was supplied by Dr. D. A. G. Galton and Dr. R. J. C. Harris, Chester Beatty Research Institute, London.

The extraction and the purification of polysaccharides from the acetone-defatted and dried material was carried out according to Harris, Malmgren and Sylvén (1954). The extraction schemes are shown overleaf.

The corresponding preparations from umbilical cords were denoted (*U–I*) to (*U–V*).

Each fraction was analysed for nitrogen (Kjeldahl), sulphur (Paulsson, 1953), phosphorus (Lowry and Lopez, 1946) and hexosamine (Blix, 1948) after hydrolysis for 24 hours in 4 M HCl at 100°C. The hexosamine was identified by ion exhcange chromatography combined with automatic recording of electrolytic conductivity according to Drake and Gardell (1952).

Determinations of the elctrophoretic mobility and sedimentation coefficient were carried out at a concentration of 0·5 per cent in phosphate buffers of pH = 7·6 and 6·8 at an ionic strength of 0·1.

### RESULTS

The analytical and physico-chemical data are given in Table I.

* Present address: Centro di Oncologia Sperimentale del C.N.R.—Sezione di Milano presso Istituto Naz. per lo Studio e la Cura dei Tumori, Milano, Piazza Gorini 22—Italy.

TABLE I.—*Analytical and Physico-chemical Data*

| | Umbilical cord polysaccharides | | | Carcinoma polysaccharides | | | Rous sarcoma No. 1 polysaccharides* | |
|---|---|---|---|---|---|---|---|---|
| | (U–I) | (U–II) | (U–V) | (C–I) | (C–II) | (C–V) | (S–I) | (S–II) |
| Nitrogen in per cent | 3·80 | 3·24 | 6·20 | 2·95 | 3·12 | 4·20 | 3·11 | 3·29 |
| Sulphur in per cent | 1·00 | 0·01 | 1·03 | 0·57 | 0·01 | 0·47 | 0·80 | 0·01 |
| Phosphorus in per cent | 0·07 | — | — | 0·10 | — | — | 0·05 | — |
| Hexosamine in per cent | 30·9 | 44·4† | 25·0 | 29·8 | 31·8 | 29·1 | 43·0 | 42·2 |
| Hexosamine in relative amount: Glucosamine | 81 | 100 | 84 | 100 | 100 | 100 | — | 100 |
| Hexosamine in relative amount: Galactosamine | 19 | — | 16 | — | — | — | — | — |
| Electrophoretic mobility × $10^5$ cm.$^2$/volt sec. at + 0·5° C. | 10·0 | — | 10·7 | 10·2 | — | 10·4 | 9·9 | 9·9 |
| Sedimentation coefficient Svedberg units | — | 1·92 | 2·35 | — | 1·01 | 1·38 | 1·08 | 0·98 |
| Optical rotation $[\alpha]^{20}_D$ | — | −35° to −40°* | | — | −53° | — | — | −44° |

* According to Harris, Malmgren and Sylvén (1954).

† Theoretical value; the values obtained are usually about 10 per cent (absolute) lower.

The total yield of polysaccharide material from the human carcinoma was about 2·5 per cent of the fat-free dry weight. Scheme 1 and Table I show that about 90 per cent of this is hyaluronic acid. The chemical composition and the electrophoretic mobility are quite similar to those of purified hyaluronic acid from human umbilical cords and from Rous fowl sarcoma. Infra-red spectroscopy (kindly performed by Dr. S. F. D. Orr) has also shown that the spectrum of preparation (*C–II*) is very similar to that of pure hyaluronate prepared from human umbilical cord and from Rous sarcoma (Orr, Harris and Sylvén, 1952). The two spectra were identical in the region 680–980 cm.$^{-1}$, indicating that the two samples have the same molecular skeleton (Orr, 1954). The sedimentation coefficient of the carcinoma polysaccharide is similar to that of sarcoma polysaccharide; however, it is lower than that of the corresponding umbilical cord preparation (*U–II*).

The sulphur-bearing constituent present in preparation (*C–I*) is completely removed by the Azure A precipitation method of Sylvén and Malmgren (1952) and is accordingly expected to be an ester sulphate polysaccharide. No galactosamine could be detected in (*C–I*) nor in (*S–I*) and the presence of chondroitin-sulphuric acid in measureable amounts, namely more than 2 per cent of total polysaccharide, is therefore excluded. The sulphur-containing substance in (*C–I*) is probably heparin or a sulphate ester of hyaluronic acid. However, none of the preparations labelled (*C–I*), (*C–II*), (*U–I*) and (*U–II*) showed antithrombic activity. This suggests that heparin is absent or that the heparin activity has been destroyed during the preparation.

The extraction of the carcinoma with detergents (Snellman) yielded an impure hyaluronic acid (*C–V*), and neither of the fractions (*C–III*), (*C–IV*) or (*C–V*) had antithrombic activity. The first two fractions were obtained in amounts too small to permit chemical analysis. The sedimentation coefficient of (*U–V*) is higher than that of (*C–V*) and the latter contains only glucosamine whereas the umbilical cord preparation (*U–V*) contains both glucosamine and galactosamine.

Extraction of the carcinoma according to Wilander (1938) yielded a sulphur-containing polysaccharide moiety (*S* content 1·04 per cent) that did not, however, exhibit heparin activity. Extraction of umbilical cords gave a similar material.

### DISCUSSION

This human undifferentiated carcinoma contained about 2·5 per cent polysaccharide material (fat-free dry weight). About 90 per cent of this was undoubtedly hyaluronate for its chemical properties which were identical with those of hyaluronic acid from human umbilical cord. The carcinoma hyaluronate, however, was of lower particle size than the umbilical. All these findings are in agreement with the observations of Warren and his co-workers (1949) and also of those of Harris, Malmgren and Sylvén (1954) for the hyaluronate of Rous fowl sarcoma,

---

Scheme 1 : Extraction and Purification Scheme of Crude Defatted Tumour Material ((*C*) 5 g.) for Polysaccharides

Extraction with 2 per cent phenol ; three times

Centrifugation

SEDIMENT discarded

SUPERNATANT

1. NaCl added up to 1 per cent.
2. Precipitation with 3 vol. ethanol.
3. Precipitate dissolved in water and successively shaken with chloroform at pH = 8, 6 and 4. Denatured proteins removed by centrifugation.
4. Digestion with ribonuclease at pH ~ 8 — 2–3 days at 37° C.
5. Dialysis against distilled water.
6. The bulk of dialysis residue lyophilized (*C–I*).

Yield 140 mg.

Addition of " Azure A " (dimethylthionine) to part of dialysis residue.

The metachromatic precipitate removed in Spinco Model L centrifuge (rotor 440 ; 60 min. at 30,000 r.p.m.

Excess of dye removed by ion-exchange resin (Dowex 50).

The filtrate lyophilized (*C–II*).

Yield ~ 90 per cent of total polysaccharide material.

indicating that mesenchymal and epithelial tumour hyaluronates are quite similar.

The sulphur-containing polysaccharide which constituted about 10 per cent of the total polysaccharide has not been identified. It contains only glucosamine and cannot therefore be chondroitin-sulphuric acid. On the other hand no antithrombic activity was observed, indicating that heparin or heparin-like substances such as the sulphate esters of hyaluronic acid, were not present in an active

---

As preliminary data indicated the presence of a sulphur containing polysaccharide, part of the crude tumour tissue (*C*) was extracted for heparin according to Wilander (1938), and part by a milder method used by O. Snellman (personal communication). The following scheme was adopted :

Scheme 2 : Extraction Scheme of Crude Defatted Tumour Material ((*C*) 3 g.) for Heparin and other Polysaccharides

Extraction in 0·5 per cent Duponol (detergent).

50 per cent saturation with $(NH_4)_2SO_4$.

Centrifugation.

Supernatant dialyzed against water.

Ba-acetate added to 3 per cent (w/v).

**Precipitate**

Dissolved in Na-salt of ethylenediamine tetra-acetic acid.

Dyalized and lyophilized.

Yield 4 mg. (*C–III*).

**Supernatant**

Ethanol added to 20 per cent by vol.

- **Precipitate**

  Dissolved in Na-salt of ethylenediamine tetra-acetic acid.

  Dialyzed and lyophilized.

  Yield 7 mg. (*C–IV*).

- **Supernatant**

  Ethanol added to 50 per cent by vol.

  **Precipitate**

  Dissolved in Na-salt of ethylenediamine tetra-acetic acid.

  Dialyzed and lyophilized.

  Yield 108 mg. (*C–V*).

state. In umbilical cord this sulphur-containing polysaccharide (perhaps a mucoitin-sulphuric acid ?) is not present in appreciable amounts since the sulphur contents of (*U–I*) and (*U–V*) correspond almost exactly with the galactosamine present. The corresponding sulphur bearing polysaccharide of Rous sarcoma also contains only glucosamine, however the sulphur content is higher and an antithrombic activity is detectable (Harris, Malmgren and Sylvén, 1954).

SUMMARY

Extraction experiments show that a human undifferentiated carcinoma, originating from the pancreas, is rich in hyaluronate which has been identified by chemical, spectroscopic and physico-chemical methods. The particle size of this tumour hyaluronate is smaller than that of normal umbilical hyaluronate, but of the same order of Rous fowl sarcoma hyaluronate.

In addition to the hyaluronic acid some unidentified sulphur-bearing polysaccharide was found, which contained glucosamine. The material is provisionally assumed to be chemically related to heparin-like substances, such as sulphate esters of hyaluronic acid, but exhibits no antithrombic activity.

The author wishes to express his sincere gratitude to Professor Arne Tiselius for the privilege of carrying out this work at his Institute, and to Dr. D. A. G. Galton and Dr. R. J. C. Harris of the Chester Beatty Research Institute, for the dried tumour material.

The author is also indebted to the Lega Italiana per la Lotta contro i Tumori, Rome, for financial support. The author wishes to thank Dr. B. Drake, Institute of Biochemistry, Uppsala, who kindly performed the quantitative separation of the amino sugars.

REFERENCES

Blix, G.—(1948) *Acta chem. Scand.*, **2**, 467.
Drake, B. and Gardell, S.—(1952) *Arkiv Kemi*, **4**, 469.
Harris, R. J. C., Malmgren, H. and Sylvén, B.—(1954) *Brit. J. Cancer.*, **8**, 141.
Kabat, E. A.—(1939) *J. biol. Chem.*, **130**, 143.
Lowry, O. and Lopex, J.—(1946) *Ibid.*, **162**, 421.
Orr, S. F. D.—(1954) *Biochem. Biophys. Acta*, **14**, 173.
*Idem*, Harris, R. J. C. and Sylvén, B.—(1o52) *Nature*, **169**, 544.
Paulsson, S.—(1953) *Acta chem. Scand.*, **7**, 325.
Pirie, A.—(1942) *Brit. J. exp. Path.*, **23**, 277.
Sylvén, B.—(1945) *Acta Radiol.* Suppl. 59.—(1949) *Ibid.*, **32**, 11.
*Idem* and Malmgren, H.—(1952) *Lab. Invest.*, **1**, 413.
Warren, G. H., Williams, E. C., Alburn, H. E. and Seifter, J.—(1949) *Arch. Biochem.* **20**, 300.
Wilander, O.—(1938) *Skand. Arch. Physiol.*, **81**, Suppl. XV.